# Melting properties by X-ray absorption spectroscopy: common signatures in binary Fe–C, Fe–O, Fe–S and Fe–Si systems

**DOI:** 10.1038/s41598-020-68244-3

**Published:** 2020-07-15

**Authors:** Silvia Boccato, Raffaella Torchio, Simone Anzellini, Eglantine Boulard, François Guyot, Tetsuo Irifune, Marion Harmand, Innokenty Kantor, Francesca Miozzi, Paraskevas Parisiades, Angelika D. Rosa, Daniele Antonangeli, Guillaume Morard

**Affiliations:** 1 Sorbonne Université, Muséum National d’Histoire Naturelle, UMR CNRS 7590, Institut de Minéralogie, de Physique des Matériaux et de Cosmochimie (IMPMC), 75005 Paris, France; 2 ESRF - European Synchrotron Radiation Facility, Grenoble, France; 3 Diamond Light Source Ltd, Harwell Science and Innovation Campus, Didcot, OX11 0DE UK; 4 Institut Universitaire de France (IUF), Paris, France; 5 Geodynamics Research Center, Ehime University, 790-8577 Matsuyama, Japan; 6 Present Address: Department of Physics, Technical University of Denmark, Kgs. Lyngby, Denmark; 7 Université Grenoble Alpes, Université Savoie Mont Blanc, CNRS, IRD, IFSTTAR, ISTerre, 38000 Grenoble, France

**Keywords:** Phase transitions and critical phenomena, Electronic properties and materials, Geochemistry

## Abstract

X-ray absorption spectroscopy (XAS) is a widely used technique to probe the local environment around specific atomic species. Applied to samples under extreme pressure and temperature conditions, XAS is sensitive to phase transitions, including melting, and allows gathering insights on compositional variations and electronic changes occurring during such transitions. These characteristics can be exploited for studies of prime interest in geophysics and fundamental high-pressure physics. Here, we investigated the melting curve and the eutectic composition of four geophysically relevant iron binary systems: Fe–C, Fe–O, Fe–S and Fe–Si. Our results show that all these systems present the same spectroscopic signatures upon melting, common to those observed for other pure late 3d transition metals. The presented melting criterion seems to be general for late 3d metals bearing systems. Additionally, we demonstrate the suitability of XAS to extract melt compositional information in situ, such as the evolution of the concentration of light elements with increasing temperature. Diagnostics presented in this work can be applied to studies over an even larger pressure range exploiting the upgraded synchrotron machines, and directly transferred to time-resolved extreme condition studies using dynamic compression (ns) or fast laser heating (ms).

The coupling of X-ray absorption spectroscopy (XAS) with laser heated-diamond anvil cell (LH-DAC) is a challenging technical development only recently implemented in synchrotron facilities^1–3^. The opportunities that stem from this combination are of great interest in physics, chemistry and planetary science.

The element selectivity and the local structure sensitivity are two of the distinctive characteristics that make XAS a very informative and multipurpose technique. Combined together, they provide the possibility of microscopic investigation of the local atomic environment of a specific element in a compound or an alloy, irrespectively of the solid, liquid or amorphous state. The X-ray absorption near edge spectroscopy (XANES) region of the XAS spectrum, also probes the unoccupied electron density of states right above the Fermi level^4–7^. Recent high pressure/high temperature absorption experiments^6,8–14^ and theoretical calculations^8,9,11,15–19^ on pure metals directly correlate changes in the XANES spectra and/or in the pre-edge to phase transitions, but to which extent these findings could be generalized to multi-component systems remains to be assessed. XANES sensitivity to the electronic structure further grants the possibility to probe the chemical environment, through relatively simple approaches like the linear combination analysis (LCA), as demonstrated in many studies at ambient conditions (Benfatto et al.^20^, among others), but never attempted under extreme high-pressure, high-temperate conditions.

In this paper, we show advanced application of XANES in combination with LH-DAC. Melting criteria are generalized and used to probe melting curves of Fe–X binary systems, where X $=$ C, O, S and Si. Furthermore, the quality of the collected data allowed us to apply LCA-based methods for data analysis, obtaining a quantitative in situ determination of chemical composition of the temperature-quenched samples under pressure, during the heating runs. The here-presented experimental protocol and data analysis offer a valuable method alternative to more classic, and not always possible, time-demanding, ex-situ analysis of recovered samples.

Binary iron alloy systems have been here chosen as test case to prove the capabilities of XANES for the experimental study of materials at high pressure and high temperature also in view of their very important implications for the understanding of planetary cores. Position in temperature and in composition of the eutectic point in a binary phase diagram anchors the reconstruction of the entire phase diagram. In the case of iron alloys, the primary constituents of planetary cores, this allows to determine which phase crystallizes upon secular cooling, yielding different crystallization paths and hence impacting the generation and sustainability of planetary dynamo^21^. Moreover, the knowledge of the chemical composition is a primordial information required to know the density contrast between solid and liquid, and thus crucial to determine whether the crystallizing solid is gravitational stable, whether it sinks or floats and to which extent crystallization might power solutal convection.

In short, XANES is a powerful tool to determine the melting curve and to obtain direct information of the electronic structure of probed materials. Some of the specific advantages of this technique are illustrated in this work by providing the measurements of the eutectic temperatures and eutectic compositions over an extended pressure and temperature range of Fe–X binary systems of primary geophysical interest, where X $=$ C, O, S and Si.

## Analysis of XANES spectra

### Melting criterion with XANES

Classically, studies investigating metals at ambient pressure and high temperature use discontinuities in the temperature evolution of the absorption signal at a single photon energy to detect solid-solid phase transitions^15^, melting and undercooling of pure substances^22–24^. This method is also referred to as T-scan method. At simultaneous high pressures and temperatures, the energy and temperature resolutions are lowered by experimental constraints such as the need for fast acquisitions and/or the micrometric sample volumes in the central portion of the laser heated hot-spot area. The sample thickness, and consequently the absorption jump in the absorption-wavelength diagram, might vary during the experiment and thus the application of the T-scan method requires an accurate normalization. As already mentioned, the loss of features in the XANES upon increasing temperature has recently been proposed and validated as melting criterion for elemental 3d metals such as iron, nickel and cobalt in experiments very similar to the ones presented in this work in terms of the covered pressure-temperature range and heating method^8–10,12,25^. In most cases results obtained by XAS experiments showed a very good agreement with those obtained by X-ray diffraction (XRD)^10,12,13^.

**Figure 1:**
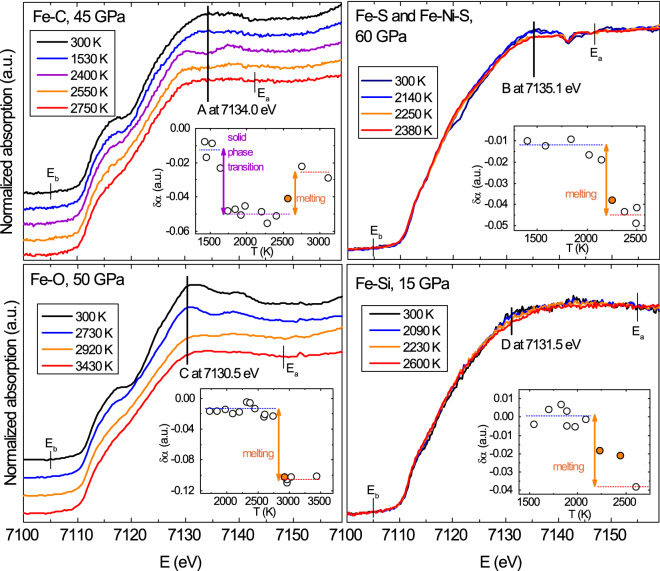
XAS spectra at Fe K-edge collected for increasing temperature showing phase transitions and melting for Fe–C, Fe–O, Fe–S and Fe–Si. The spectra in black are at ambient temperature, the blue are solids, the violet (in the Fe–C system) is a solid with a different structure, the orange spectra are first appearance of liquid and the red are liquids. For clarity, sequences of spectra in the left panel are vertically shifted, while in the right panel they are superimposed. The insets show for each of the four binary systems the change of white line peak $\delta \alpha$ at the specific energies indicated with letters A, B, C and D respectively. Mixed phases are indicated with orange circles. A discontinuity in the single energy X-ray absorption is an indication of solid-solid phase transition (as for Fe–C) or melting. Horizontal dashed lines are a guide for the eyes.

Pure Fe melts from a solid with a face-centered cubic (fcc) structure over a wide pressure range, from 15 to about 100 GPa^13,26^. Melting from fcc structure over a comparable pressure range is common for all late 3d transition metals, i.e. metals with 3d orbitals more than half filled. The melting criterion in these systems is the flattening of the XANES shoulder and the disappearance of the first two oscillations after the edge, typical of an fcc structure. We observe that this melting criterion is also valid for the Fe–C and Fe–O systems (see left panel of Fig. 1 and “Methods” section for details on XAS data collection and normalization). We also note that in the investigated pressure range, Fe–C exhibits, before melting, a temperature-induced solid–solid phase transition, evidenced by a clear change in the XANES features at around 7130–7140 eV (violet spectrum in the top left panel of Fig. 1). The nature of this solid–solid transition will be discussed in the “Results” section. The high-temperature solid phase melts upon heating at 2550 K. Melt in the Fe–O system was observed at 50 GPa at 2920 K through a clear loss of features in the XANES (Fig. 1). XANES spectra collected on Fe–S and Fe–Si systems, when compared to Fe–C and Fe–O, show fewer features and less clear variations with increasing temperatures (right panel of Fig. 1). The solid–liquid transition is however still distinguishable from the flattening of the XANES features around the energies identified with B and D in Fig. 1. We therefore applied the T-scan method^12,15,22–25^ as a second criterion to double-check estimated melting temperature. In the inset of Fig. 1 we plot the variation of the absorption coefficient at a specific energy as a function of temperature, hereby called $\delta \alpha$ following the nomenclature in Di Cicco et al.^24^. The transition from the solid to the liquid state leads to a discontinuous variation and an abrupt change of XANES features due to the loss of the long-range and medium-range order upon melting. In the insets of Fig. 1 the $\delta \alpha$ ($\hbox {A} = 7134.0$ eV for Fe–C, $\hbox {B} = 7135.1$ eV for Fe–S, $\hbox {C} = 7130.5$ eV for Fe–O and $\hbox {D} = 7131.5$ eV for Fe–Si) shows clear discontinuities that are associated to melting for the four binary systems. The energy for the T-scan method is chosen in order to maximize the contrast in the $\delta \alpha$ as a function of temperature. This value depends on the atomic structure of the last solid and the difference in the electronic density of states between the hot solid and the liquid^25^.

The same changes observed in the XANES in this work were related to melting in previous experiments conducted on pure late transition 3d metals such as iron^8–10^, nickel^12^ and cobalt^25^ and recently on Fe-Ni^27^. In the case of cobalt, ab-initio calculations showed that such changes are due to the appearance of multiple configurations proper of the liquid phase and associated to the loss of crystalline order. The electron density of states also varies at the energies where discontinuous changes are detected. The loss of XANES features, together with discontinuities in the $\delta \alpha$, can thus be used as a generalized melting criterion for late 3d transition metal based systems.

In the present study the adopted melting criterion was further verified through textural and chemical analysis of several recovered samples using the focused ion beam (FIB) and scanning electron microscope (SEM) approach detailed in Morard et al.^28^. An example will be discussed later in the “Results” section and illustrated in Fig. 4.

### Composition determined in situ by XANES

**Figure 2:**
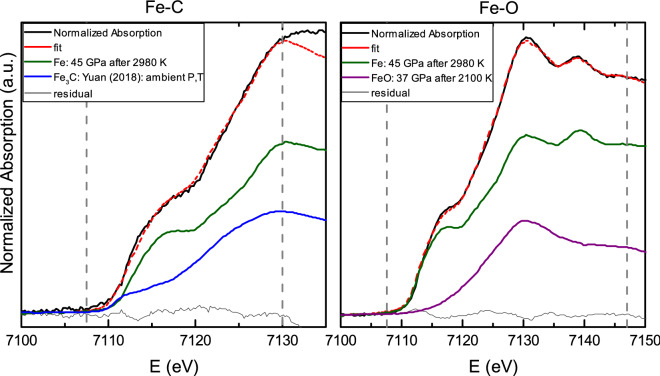
Absorption spectra at Fe K edge measured for the iron-bearing binary systems can be well reproduced by linear mixing of reference spectra of the corresponding end members, allowing the quantification of the amount of light element in each binary system. Example of linear combination analysis (LCA) of Fe–C (left) and Fe–O (right). Reference spectra of stoichiometric end-members are also represented ($\hbox {Fe}_3\hbox {C}$ reference is from Yuan et al.^29^, Fe is from Morard et al.^13^ and FeO is from Boulard et al.^31^), and their intensities are proportional to their weight resulted from the fit. Being $\hbox {E}_0 = 7112$ eV, the range for the fit was 7107–7130 eV for the Fe–C system and 7107–7147 eV for the Fe–O system, as indicated by dashed vertical lines. This range is chosen in order to have the best fit quality within the largest energy range. In the case of Fe and FeO the reference spectra are temperature-quenches collected after heating at the indicated temperature.

As already mentioned, the XANES signal, due to its sensitivity to the electronic structure of the absorber, contains information about its chemical environment. The chemical composition of the sample can thus be determined by proper analysis of the spectra collected on the temperature-quenched samples, provided that XANES of reference compounds are available^20^ (and preferably at close P-T conditions). In Fig. 2, the XANES spectra of pure Fe and stoichiometric $\hbox {Fe}_3\hbox {C}$ and FeO are compared to the ones obtained in the binary Fe–C and Fe–O systems.

To quantify the relative amounts of end-members in Fe–C and Fe–O liquid binary systems , we used a linear combination analysis (LCA)^20^ employing the program ATHENA^30^. In this approach, the normalized spectra of temperature-quenched samples are fitted to a theoretical curve obtained as linear combination of the normalized spectra of stoichiometric samples.

A set of high-quality reference spectra at the relevant pressures is of crucial importance for obtaining reliable results. First of all because pressure can induce phase transitions and secondly, even when no transitions occur, the effect of pressure is to shift the EXAFS and the XANES features to higher energies. This shift was shown to start gradually few eV higher than the edge energy^25^, therefore it is quite small in the edge region, while it becomes important in the EXAFS domain. Here a short range for the fitting around the edge has been chosen so to minimize any error due to small pressure offsets between the analyzed spectrum and the reference.

Reference spectra for iron and stoichiometric FeO at ambient temperature, quenched after heating at different pressures, were collected in this study and during previously published experiments (iron^13^ and FeO^31^). Due to the absence of high-pressure references, for the analysis of $\hbox {Fe}_3\hbox {C}$ we used XANES measured at ambient conditions^29^. We note however that for the exhamined compositional space of the Fe–C system and in the chosen energy range, the differences in the near edge features between the two end-members appear to be largely dominant in respect of the pressure variations of $\hbox {Fe}_3\hbox {C}$ XANES spectra, thus allowing to perform LCA analysis even though missing a set of stoichiometric $\hbox {Fe}_3\hbox {C}$ spectra at relevant pressures.

An example of fit performed with LCA is shown in Fig. 2, together with the used reference spectra. Starting from the end-member proportions obtained from LCA, it is a straight exercise to quantify the eutectic composition, and specifically the amount of light elements in the eutectic liquid of the Fe–C and Fe–O iron binary systems, using the known content of carbon and oxygen in stoichiometric $\hbox {Fe}_3\hbox {C}$ (6.7 wt% of carbon) and in stoichiometric FeO (22.3 wt% of oxygen).

## Results

### Eutectic melting curves

**Figure 3:**
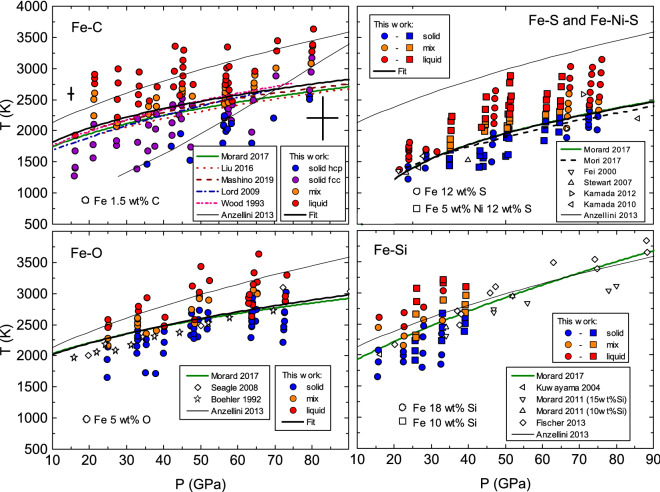
Eutectic melting curves of four Fe–X binary systems are presented and compared to the literature: Fe–C^28,36,39,44,45^, Fe–O^28,35,37^, Fe–S^28,32,34,40–42^ and Fe–Si^28,33,38,43^. Uncertainties on pressure and temperature determination are represented as black crosses for low and high P-T data points in the Fe–C system and are equivalent for all four binary systems at comparable pressure temperature conditions. For reference, the melting curve of pure Fe as measured by XRD by Anzellini et al.^26^ and recently confirmed by XAS^13^ is shown as thin black line. The thick black curves represent a Simon–Glatzel fits to data points for which the first appearance of the liquid was observed in this study through XAS together with points from selected studies in literature (see main text for extended discussion). Fit parameters are reported in Table 1.

All the investigated pressure–temperature points for the four different binary systems are shown in Fig. 3. Following the same color code as in Fig. 1, blue and violet spectra represent solid phases, orange spectra highlight the onset of melting and red spectra show liquid phases. In the case of Fe–S and Fe–Si, two slightly different starting compositions were used and are distinguished in Fig. 3 as circles and squares.

In all the four cases we observe an overall good agreement, within the pressure and temperature uncertainties, between our results and previous works, independently of the sample environment, the adopted detection technique or the starting composition. Over the pressure range of interest in this study, samples are most commonly pressurized and heated with LH-DAC, or multi anvil press for the lower pressures^32–34^. Melting detection methods include visual observation of sample surface movements^35^, the plateau in the temperature versus laser power plot^36^, appearance of liquid diffuse scattering in XRD measurements or disappearance of the solid peaks^28,37–44^, and ex situ textural analysis (FIB coupled with SEM or microprobe) on recovered quenched samples^28,32,34,40,44^. Starting compositions vary for Fe–C between 1.5 and 4.0 wt% C, for Fe–O between 1.2 and 11 wt% O, for Fe–S between 4 and 16.1 wt% S and for Fe–Si between 10 and 18 wt% Si. Same melting temperature for a system with different starting compositions is expected for incongruent melting, where the obtained liquid is at the eutectic composition.

Considering each individual system more in detail, in the top left panel of Fig. 3, blue and violet symbols indicate two different solid phases of Fe–C. Analysis of XANES suggests that the low-temperature phase (in blue) is dominantly hexagonal close packed (hcp) and the high-temperature phase (in violet) is dominantly fcc. The pressure–temperature conditions of this hcp to fcc phase transition are, within the error bars, compatible with the ones reported by Anzellini et al.^26^ for pure Fe (thin black line in the figure), suggesting that the temperature induced hcp–fcc solid–solid transition is only slighted affected by C doping. In contrast, the melting temperature is significantly lowered by the presence of carbon. We compiled present XAS results and most recent literature results^28,44^ and fit the whole data set to a Simon–Glatzel equation. The resulting melting curve is represented as a thick black curve in Fig. 3.

XAS data of Fe–O shown in the bottom left panel of Fig. 3 are in very good agreement with the most recent measurements^28^ while a slight discrepancy can be noticed with the earlier data^35, 37^. The joint Simon-Glatzel fit of our new data together with previous XRD data^28^ results in the thick black curve that, in the range under analysis, is virtually indistinguishable from that from Morard et al.^28^ (green thick curve).

In the top right panel of Fig. 3 the melting curves obtained in the Fe–S system, with and without the addition of 5 wt% of Ni, are compared (Fe-12 wt% S and Fe-5 wt% Ni-12 wt% S are represented with circles and squares respectively). The obtained melting temperatures are similar for both compositions, suggesting that the addition of 5 wt% of nickel has negligible effect on the melting temperature within current experimental uncertainties, which is in agreement with recent findings on the Fe-Ni melting curve^27^. The difference of about 125 K at 20 and 40 GPa previously reported by Stewart et al.^34^ is indeed within present uncertainties and could therefore not be resolved. The melting curve for the Fe–S system, represented with a thick black curve in Fig. 3, was obtained by fitting a Simon–Glatzel equation to all our data points, irrespectively of the addition of nickel, together with data from Morard et al.^28^ and Mori et al.^40^, the most recent literature results. The resulting melting curve is virtually indistinguishable from the one reported by Morard et al.^28^. Data from Fei et al.^32^, Stewart et al.^34^ and Kamada et al.^41,42^ are scattered around the fitted curve, thus in agreement within the uncertainties. Simon–Glatzel fit parameters for these three binary systems are reported in Table 1.

Previous studies reported a dependency of the melting temperature on the starting composition in the Fe–Si binary system, arguing for a eutectic behavior. However, in the present study we could not resolve a difference over the investigated pressure range (up to 40 GPa) for starting composition with 18 wt% and 10 wt% Si shown as circles and squares in Fig. 3 respectively. In view of the limited pressure range covered in this study, we did not attempt to fit the melting curve, but we stress that our melting temperatures are in very good agreement with melting curve proposed by Morard et al.^28^ based on previous results^38,43^.

Out of the iron binary systems investigated in the present work, only Fe–Si exhibits a melting temperature comparable to that of pure iron. In contrast, the addition of O, C and S significantly lowers the melting temperatures, with differences reaching 500 K (O), 750 K (C) and 1000 K (S) at 90 GPa.

**Table 1:** Fit parameters of Simon–Glatzel equation: $T_m=T_0 [(P_m-P_0)/a+1]^{1/c}$.

	$\hbox {T}_0$ (K)	$\hbox {P}_0$ (GPa)	a	c
Fe–C	1420	0	5.9	4.1
Fe–O	1800	0	21.6	3.3
Fe–S	1260	21	10.3	3.0

### Eutectic compositions

**Figure 4:**
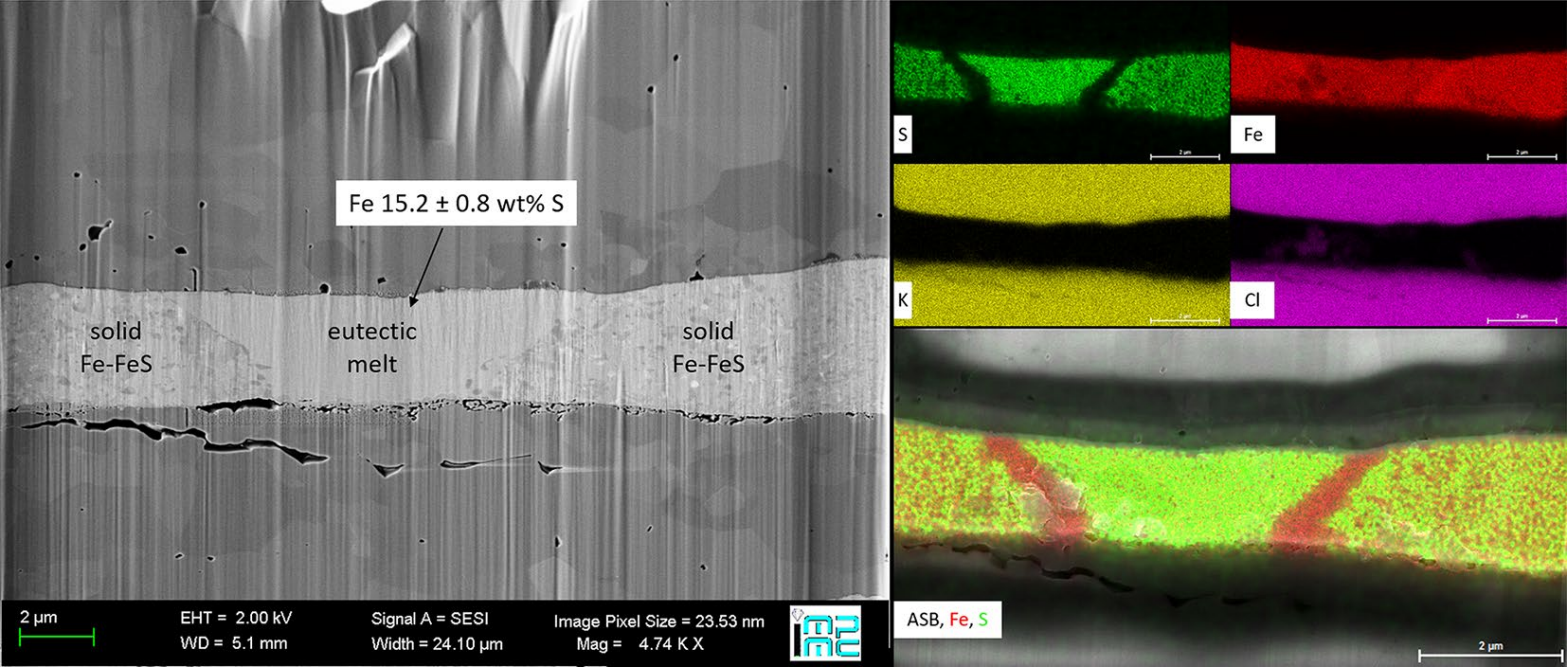
Image on secondary electron secondary ion (SESI) detector (left panel) and elemental concentration maps from EDX-SEM (energy dispersive X-ray - scanning electron microscope) analysis (right panel) of a transverse section of quenched Fe–S sample of Fe-12 wt% S starting composition, recovered after laser heating at $34\pm 3$ GPa and $1730\pm 120$ K. Visible in the SESI image are the sample are the Fe–S sample (center, light grey area) and two KCl disks at the top and the bottom (dark grey). Clear textural differences can be distinguished in the sample. The homogeneous texture in the central area can be associated with a quenched liquid coexisting at high temperature with a solid formed at the rim of the laser heated spot (heterogeneous textures). The concentrations are shown for S (green), Fe (red), K (yellow) and Cl (violet) in the EDX-SEM maps. Chemical maps allow excluding melt contamination by K or Cl. Preferential liquid–solid partitioning of sulfur upon melting led to its depletion in the solid formed at the melt pool border.

The composition of the eutectic liquid was derived from XAS data obtained in situ at high pressures on samples quenched after melting and ex situ by microprobe measurements on recovered, FIB cut samples. In the case of ex situ chemical measurements we also determined the composition of the solid phase coexisting with the eutectic liquid. The compositions of the liquid and its temperature-quench are assumed to be the same. Laser heating in a DAC induces significant thermal gradients in the sample that also lead to chemical diffusion of elements, the so-called Soret effect^46^. A liquid pocket forms at the center of the hotspot, surrounded by a coexisting solid phase (Fig. 4). These phases are in chemical equilibrium due to the fast diffusion coefficients at high temperatures for the elements present in the investigated systems. With increasing temperature above the eutectic point, the liquid portion will grow in size but will not significantly change in composition. This phenomenon was reported in previous LH-DAC studies on binary Fe–X systems^28,40,44,47^.

**Figure 5:**
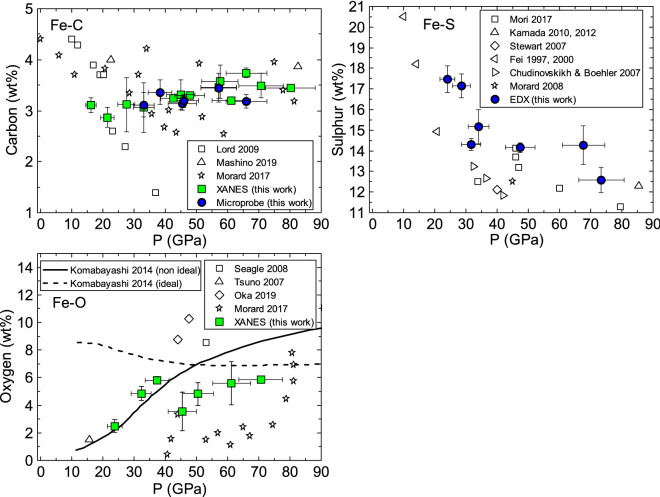
Eutectic liquid compositions from in situ XANES diagnostics on temperature-quenched samples ex situ and/or analysis techniques (microprobe analysis and EDX-SEM), compared to those of the literature for Fe–C^28,36,44^, Fe–O^28,37,47–49^ and Fe–S^32,34,40–42,50–52^. XANES points are the average of the results from the analysis of several temperature-quenched data after melting at similar conditions. When the pressure of the spectrum to analyse is in between the pressure for which reference spectra were available, the chemical evaluation was performed with both lower and higher pressure references and the result averaged. The error bar is evaluated as the variance between the different results of analysis from individual spectra, as shown in the Supplementary information.

For the Fe–S system, recovered samples were analyzed with EDX-SEM in order to check chemical contamination and for textural analysis (see Fig. 4). A global decreasing trend in S content in the eutectic liquid with increasing pressure was identified, with the actual S amount slightly higher compared to previous studies^32,34,40–42,50,53^. The Fe–Si binary system is expected to melt with a 1:1 solid-liquid partitioning^54,55^, so it was not considered in the following analysis.

Literature results for the Fe–O binary system show a large scatter of the liquid composition. The difference in the two most recent studies^28,47^ can be rationalized by C contamination in Morard et al.^28^. Our estimations, obtained by analysis of XANES data based on the available appropriate XANES references for both the end-members at high pressure, are in between, and show a trend compatible with a progressive increase in O content in the eutectic liquid with increasing pressure, as suggested by the non ideal model by Komabayashi et al.^48^. For the Fe–O binary system, samples were not recovered.

In the case of Fe–C, the relative proportion between two end-members Fe and $\hbox {Fe}_3\hbox {C}$ is evalued in situ with LCA analysis and the total amount of carbon in the sample is obtained from the derived proportion of $\hbox {Fe}_3\hbox {C}$ phase plus the amount of carbon in solution in solid iron, which was estimated according to Mashino et al.^44^. Details on this calculation are presented in Supplementary information. For comparison, carbon content of the quenched samples was also evaluated ex situ by EDX-SEM. The eutectic liquid compositions obtained for the Fe–C binary system with XANES is in excellent agreement with those from EDX-SEM and with estimations from thermodynamics calculations by Fei and Brosh^56^. The eutectic liquid composition is observed to slightly evolve with pressure, in agreement with recent studies^28,44^, but in opposition to the strong negative trend proposed in early work^36^.

Textural analysis independently confirmed melting detection by XANES, but even more relevant is the clear agreement between ex situ and in situ eutectic composition measurements obtained in the present study (Fig. 5).

## Discussion

The present work highlights the versatility and capability of XANES when applied in combination with laser heated diamond anvil cells. We demonstrate that solid–solid and solid–liquid phase transitions are unambiguously revealed by distinct changes in XANES features as a function of pressure and temperature (Fig. 1).

The criteria presented in this work for the solid–liquid phase transition are proved to be common not only to late 3d transition metals (Fe, Co, Ni, Cu)^10,12,13,23–25^, but also to several Fe–X systems under extreme conditions of pressure and temperature. Due to the structural and electronic similarities among the late 3d transition metals, we expect this criterion to be valid for a large number of late 3d transition metals bearing binary systems.

**Figure 6:**
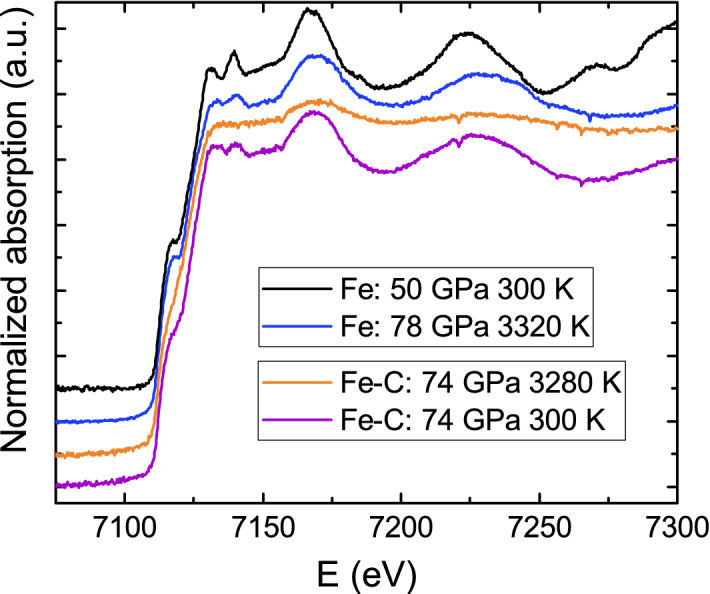
Comparison of the XANES spectra at Fe K-edge of molten Fe–C, solid iron at similar pressure and temperatures and a solid iron at room temperature. The loss of features in the Fe–C XANES is a consequence of melting and cannot be ascribed to the damping of the oscillations due to the increase of temperature.

Focusing on in situ melting diagnostics, XAS can be used as a complementary technique to XRD. Both XAS and XRD are bulk techniques that allow to precisely track changes in material’s properties, in contrast to the visual observation of the sample surface. In XRD, melting is related to the appearance of the diffuse scattering: the diffraction peaks disappear due to the loss of long range order, and are replaced by diffuse scattering signal, which contains information about the short range ordering. The changes detected in the XANES are also associated to intrinsic physical changes resulting from the phase transition from the solid to the liquid^25^: the smearing/loss of features at the absorption edge can be understood as the consequence of the coexistence of multiple configurations in the liquid phase, directly related to the modification of the electronic bands around the Fermi energy. These changes are in principle independent of the specifically considered system and thus give universality to the melting detection method. As the atomic vibrations become more important the higher is the temperature, the mean square relative displacement (Debye–Waller) increases with temperature, causing the damping of the EXAFS oscillation, in particular at higher energies. This oscillations damping is clearly visible when comparing the measured Fe K-edge spectra of solid iron at room temperature and at high temperature (Fig. 6). Differently, in a solid–liquid transition, sudden changes are visible at low energies, in the XANES region, while oscillations at higher energy still remain. The comparison between solid Fe and liquid Fe–C at very similar pressure and temperature (also shown in Fig. 6) provides a further confirmation that the XANES changes attributed to melting are clear indication of solid-liquid phase transition and are not due to temperature increase. The oscillations still visible in the EXAFS contain information on the interatomic distance in the liquid phase, and can thus be further exploited^14^. However, because of the limited energy range and the data quality, the information extracted remains limited to the first neighbor distance. Thanks to the Extremely Brilliant Source (EBS) upgrade^57^ with consequent renovation of the ID24 beamline^58^, in the near future it will be possible to perform absorption measurements in LH-DAC over an even longer energy range in the EXAFS ($\sim 1000$ eV) and using a much smaller X-ray beam ($\sim 1$ $\mu$m full width half maximum, FWHM). Better data quality and more extreme conditions will be achieved allowing to reveal the structure of melts at planetary core conditions. In parallel, characterization of structural transformations using a supervised machine learning method, namely, artificial neural network, is ongoing, in order to extract information on the local structure starting from both XANES and EXAFS^59,60^. We believe that the combination of high quality data and the use of more sophisticated methods for the analysis will allow to fully explore all the structural and electronic information hidden in the XANES.

**Figure 7:**
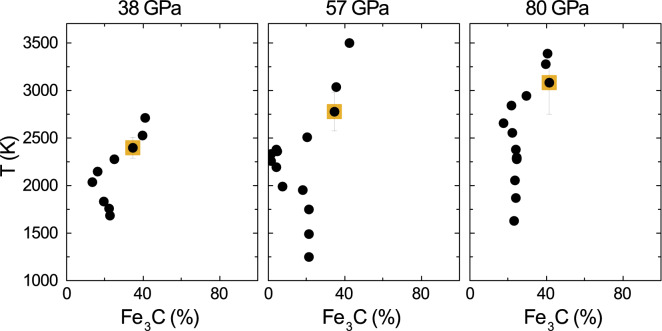
$\hbox {Fe}_3\hbox {C}$ proportion derived using LCA on XANES measured on temperature-quenched samples, laser heated at the temperature indicated in the vertical axis. In the three cases, for three temperature runs at 38, 57 and 80 GPa, the starting $\hbox {Fe}_3\hbox {C}$ proportion is $\sim 22$ wt%, corresponding to $\sim 1.5$ wt% C, well compatible with nominal composition and electron microprobe analysis of starting material. The decrease in the $\hbox {Fe}_3\hbox {C}$ content at increasing temperature can be interpreted as the progressive dissolution of carbon in solid iron. The total amount of carbon has to be evaluated as the carbon in the $\hbox {Fe}_3\hbox {C}$ phase plus the carbon in solid solution with Fe, as shown in Supplementary information. The yellow square in each run indicates the temperature at which the onset of melting was detected: this is the eutectic point (eutectic temperature and eutectic composition).

XANES measurements can be used to track changes in physical state as a function of temperature by analysis of the temperature quenches after each pulsed heating (here 1 s). LCA analysis of XANES spectra can be also used to obtain compositional information on the probed materials, including the critical case of quenched liquids (Fig. 2). The agreement between the eutectic liquid composition from in situ determination by XANES and from classical ex situ techniques such as EDX-SEM and electron microprobe, together with the overall agreement with previous literature, clearly shows the suitability and great potential of XANES as in situ probe of the eutectic melt composition at extreme conditions, complementary to the more time-consuming FIB ex situ analysis techniques (Fig. 4). Application of XANES is particularly relevant for light elements, difficult to be quantified in situ by other techniques such as XRD (whose sensitivity to the grain size and orientation hinders a quantitative analysis when approaching the melting temperature), and when samples cannot be recovered. Figure 7 shows the results of LCA analysis at three different pressures. The $\hbox {Fe}_3\hbox {C}$ proportion was evaluated in situ on temperature-quenched samples after pulsed heating at the temperature reported in the y-axis. The decrease of $\hbox {Fe}_3\hbox {C}$ proportion while increasing temperature is compatible with the dissolution of carbon in solid iron^44^. Upon further temperature increase, the sample melts and the carbon content equilibrates to the eutectic composition. The carbon content can be evaluated as the one in the $\hbox {Fe}_3\hbox {C}$ phase added to the one known to be in solid solution, as discussed in the Supplementary information. LCA method shown in this work allows to determine both the structural rearrangement and the composition in situ, thus tracking the pressure evolution of the eutectic point (yellow square in Fig. 7), which is a critical information to build phase diagram and to establish accurate thermodynamic models of the investigated systems.

In Aprilis et al.^61^ it was shown that chemical reactions of iron samples with diamonds to form iron carbide are independent of the continuous or pulsed nature of the laser heating, at least for a pulse duration down to $2\, \mu \hbox {s}$. Here we show that upon laser exposures of 1 or 2 s, chemical variations are detectable simultaneously with the phase transition. Reducing the laser pulse to shorter duration than the microseconds scale would allow identifying if there is a threshold in the pulse duration under which structural rearrangement or phase transitions happen faster than chemical changes. This is only possible thanks to the fast acquisitions proper of energy dispersive XAS.

Moreover, XAS is very suitable for measurements on iron-bearing samples at high pressure, where the thickness maximizing the signal to noise ratio ideally matches the LH-DAC requirements to minimize axial thermal gradients^62^. An initial sample thickness of 4 or $5\, \upmu \hbox {m}$, allows to simultaneously determine the phase diagram and study the compression of the system in the liquid state. In the case of XRD, a good quality diffuse scattering from a liquid signal requires a thicker sample, and heating above melting temperature is often necessary to achieve large melting fraction versus remaining solid portion. Practically, two different sample thicknesses have to be chosen in XRD experiments dedicated to phase diagram determination (thinner samples) or for the study of the liquid properties (thicker samples).

## Conclusion

We report a systematic study on melting curves of iron-rich binary systems performed by X-ray absorption spectroscopy in combination with laser heated diamond anvil cell. Our experimental results allow confirming and generalizing the melting criterion, establishing the signature of melting in the XANES region common to late 3d metals and iron alloys. Specifically, the eutectic melting of iron alloyed with C, O, S and Si was determined and compared with the literature. The consistency between our in situ results by absorption spectroscopy, ex situ results by electron microprobe analysis, and the literature provided a further proof of the suitability of this technique to detect melting and allowed to refine melting curve and to update Simon–Glatzel fit parameters for Fe–C, Fe–O and Fe–S eutectic systems. We exploited the capability of XANES to probe both the atomic and the electronic structures, which are characteristic for each system, to track in situ changes in physical state together with changes in chemical composition of the investigated samples. In particular XANES have been used to quantify the variation of light elements content during the heating run and after complete melting. Accordingly, XANES can be used as complementary technique to XRD to measure the pressure evolution of the eutectic point (temperature and composition). We expect this approach to be used in future static or dynamic compression studies of iron binary systems, profiting of the recent development in the X-ray spectroscopy field^9,11^. Linear combination analysis might be particularly important for pulsed laser heating, since it allows tracking possible changes in composition with temperature, and in all cases where samples cannot be recovered, such as in most of the dynamic compression experiments.

## Methods

### Starting materials

Several binary systems have been investigated in this work, with nominal composition: Fe-1.5 wt% C, Fe-5 wt% O, Fe-12 wt% S, Fe-5 wt% Ni 12 wt% S, Fe-18 wt% Si and Fe-10 wt% Si. The Fe–O samples were synthesized by the DEPHIS Company, sputtering iron onto a glass slide under $\hbox {O}_2$ flow. The other samples were prepared by induction melting at the Institut de Chimie et des Matériaux de Paris-Est (ICMPE) in Thiais, France. The resulting samples, with a sub-micrometric texture and a thickness between 20 and $50 \,\upmu \hbox {m}$, were precompressed between two diamonds down to about 4 or $5\, \upmu \hbox {m}$. The samples used for this work were from the same batches as the ones previously used in XRD experiments by Morard et al.^28,38^. For all samples, chemical analysis confirmed the nominal composition within the error bar.

### In situ laser heating XAS experiments in diamond anvil cell

XAS experiments were performed at the energy dispersive beamline ID24 at the European Synchrotron Radiation Facility (ESRF). In the energy dispersive geometry the incident pink X-ray beam is dispersed by an elliptically bent Si (111) crystal and then focused to about 4x5 $\upmu \hbox {m}^2$ (vertical $\times$ horizontal) FWHM on the sample position. XAS signal was measured for all the samples at the Fe K-edge 7112 eV. A range in energy between 7000 and 7500 eV allowed to record both X-ray absorption near edge spectroscopy (XANES) and extended X-ray absorption fine structure (EXAFS). The X-rays transmitted by the sample were recorded on a position sensitive detector, a FReLoN camera with an Hamamatsu chip^63^, where the position was correlated to the energy using an iron standard reference.

High pressure conditions were obtained using membrane driven DAC equipped with either single crystal or nanopolycrystalline^64^ diamonds, having culet diameters ranging from $150/300\, \upmu \hbox {m}$ (beveled diamonds) to $350\, \upmu \hbox {m}$ and a thickness of about 1.6 mm. $200 \,\upmu \hbox {m}$ thick rhenium gaskets were pre-indented to a thickness of about one tenth of the culet size. An hole of one third of the culet diameter was laser drilled in the center of the pre-indentation to serve as sample chamber. The samples were then loaded in the DAC embedded in previously dried KCl disks. These disks were prepared at the Institut de Minéralogie, de Physique des Matériaux, et de Cosmochimie (IMPMC, Paris) by compressing KCl powder to the target thickness and then laser cut with a femtosecond-laser to the needed diameter as described in Ref.^13^. Samples were heated from both sides using the laser heating system installed at the ID24 beamline of the European Synchrotron Radiation Facility (ESRF)^3^, equipped with two CW Nd:YAG lasers (IPG photonics) of $1064 \,\upmu \hbox {m}$ wavelength. Radial temperature gradients were reduced by slightly defocusing the lasers to a hot-spot size diameters of $\sim 20 \,\upmu \hbox {m}$ FWHM (this size was 3–4 times the size of the focused X-ray beam diameter). It should also be noted that the optimal initial sample thickness for iron binary systems is of few microns, which naturally matches DAC constraints for experimentation at Mbar pressure and minimizes the axial thermal gradients. As shown in Fig. 4, the sample thickness under pressure does not exceed 3–4 $\mu \hbox {m}$, granting temperature gradients below 200 K^62^, comparable to the error bars of spectroradiometric measurements.

At the target pressure, the sample was heated for about 1.1 up to 2.1 s at a selected laser’s power. During this time the black body radiation emitted from the heated sample was collected using the beamline spectrometer and the temperature was measured by fitting the obtained black body radiation with a Planck function in the grey body approximation. Simultaneously, X-ray absorption spectra were collected on the FReLoN detector with an exposure time of about 100 ms per spectrum. The final XAS signal was obtained by averaging about 10–20 spectra to improve the signal to noise ratio. XAS spectra were collected at ambient temperature on the heated sample area after each heating pulse in order to monitor sample modifications and to identify potential chemical reactions. The reported error in the temperature corresponds to the standard deviation of the two-color fit as in Benedetti et al.^65^. The pressure was measured before and after each heating run by ruby (Cr:Al$_{2}$O$_{3}$) fluorescence method, using the non-hydrostatic calibration by Mao et al.^66^. The thermal pressure correction was applied to the data as in Boccato et al.^12^. Overall, error bar on pressure is of $\sim 10\%$ of the pressure value.

More details about the measurement strategy and pressure and temperature metrology for these experiments are provided in Refs.^12,13^.

### Normalization of XANES spectra

For the analysis of XAS data obtained in this work we first normalized the raw absorption spectra using two distinct energy points. The first point was located below the absorption edge (here referred to as Eb) and was set to zero. The second point was chosen after the absorption edge (here referred to as Ea) and was set to one. Eb is 7105.0 eV for the four systems, while Ea is 7142.8 eV for Fe–C, 7148.4 eV for Fe–O, 7146.1 eV for Fe–S and 7145.6 eV for Fe–Si, with a variability of about 0.1 eV due to the pixel to energy conversion in the different experiments. Eb and Ea are shown in Fig. 1 for the four systems. The choice of Ea, depends on the structure of the specific binary system. The two points have to be chosen such as their intensity is unaffected by the Debye Waller damping caused by the temperature increase.

### Analysis of recovered samples

Several Fe–C and Fe–S samples were recovered for additional ex situ analysis after quenching from high pressure and temperature conditions. Fe–O and Fe–Si could not be recovered. A cross section was cut through the laser heated area using a focused ion beam (FIB) milling. Textural analysis was then performed using a Zeiss Neon40ESB SEM. The chemical composition determined by both electron microprobe and energy dispersive X-ray spectroscopy (EDX) elemental mapping in the SEM.

The carbon content of Fe–C recovered samples was determined using the Cameca SX100 electron microprobe at CAMPARIS, Sorbonne Université, using pure Fe and stoichiometric $\hbox {Fe}_3\hbox {C}$ as standard references. A cold catcher, cooled down with liquid nitrogen, and a micro-leak of oxygen were used to minimize the carbon contamination during the analysis. Operating conditions were 10 kV and 10 nA for a counting time of 45 s on peak and 20 s on background. To ensure electrical contact with the sample holder, the samples were coated with 3 nm of platinum (plasma coating for 10 s with a current of 60 mA). Carbon coating was avoided to prevent bias in the carbon quantification. To ensure the stability of the calibration, pure iron was measured before and after each measurement on the sample.

The sulphur content in the Fe–S platinum coated samples was determined by EDX analysis on the SEM FEG Zeiss Ultra55 (IMPMC, Paris), using a 15 keV electron beam and measuring the emitted intensity on a silicon drift detector (SDD). The intensity calibration was performed on a copper reference sample. For quantitative analysis we used the Phi-Rho-Z method using pure iron and natural pyrite ($\hbox {FeS}_2$) as standard references. This method allowed to deconvolute the Fe and S peaks in natural pyrite and therefore provided an accurate quantification.

## Supplementary information

Supplementary information.

## Data Availability

The datasets generated during and/or analysed during the current study are available from the corresponding author on reasonable request.
