# Supplementary material for: Melting properties by X-ray absorption spectroscopy: common signatures in binary Fe–C, Fe–O, Fe–S and Fe–Si systems
Source: Sci Rep. 2020 Jul 15;10:11663. doi: 10.1038/s41598-020-68244-3 (PMC7363681; doi:10.1038/s41598-020-68244-3)
Supplement: Supplementary file 1 — Supplementary information. [file 41598_2020_68244_MOESM1_ESM.pdf]

Supplementary Material
Melting properties by x-ray absorption spectroscopy: common signatures in binary Fe-C, Fe-O, Fe-S and Fe-Si systems

Silvia Boccato[1,2,*], Raffaella Torchio[2], Simone Anzellini[4], Eglantine Boulard[1], François Guyot[1,5], Tetsuo Irifune[6], Marion Harmand[1], Innokenty Kantor[2,3], Francesca Miozzi[1], Paraskevas Parisiades[1], Angelika D. Rosa[2], Daniele Antonangeli[1], and Guillaume Morard[1,7]

[1]Sorbonne Université, Muséum National d'Histoire Naturelle, UMR CNRS 7590, Institut de Minéralogie, de Physique des Matériaux, et de Cosmochimie (IMPMC), 75005 Paris, France
[2]ESRF - European Synchrotron Radiation Facility, Grenoble, France
[3]Present affiliation: Department of Physics, Technical University of Denmark, Kgs. Lyngby, Denmark
[4]Diamond Light Source Ltd, Harwell Science & Innovation Campus, Didcot, OX11 0DE, United Kingdom
[5]Institut Universitaire de France (IUF)
[6]Ehime University, Matsuyama, Japan
[7]Present affiliation: ISTerre, Université Grenoble Alpes, Grenoble, France
[*]silvia.boccato@upmc.fr

The supporting information includes detailed description of analysis missing in the main text.

1 Light element content determination with LCA

X-ray absorption near edge spectroscopy (XANES) allows to perform an *in situ* determination of the light element content. In case of co-existence between two phases the linear combination analysis (LCA) allows to evaluate the proportion between the end-members by performing a fit of the data to a linear combination of the normalized end-members XANES reference spectra. As widely discussed in the main text, this analysis was performed for Fe-C and Fe-O systems, producing excellent results. However, critical aspects of this technique applied two our data raised during the analysis, and the assumptions made deserve to be presented and discussed.

- Light elements in solution

In case of a mix of phases, LCA is sensitive to the ratio between constituent phases. With the reference XANES spectra of Fe and FeO it is possible to determine the relative weight of the two phases in an Fe-O system. Assuming that all the oxygen is contained in the FeO phase and knowing that in FeO the oxygen content is 22 wt%, the total oxygen content in Fe-O is proportional to the FeO weight. This assumption, though, is not valid in Fe-C systems, where it is known[1] that the Fe_3C phase is not the only reservoir of carbon. Up to 2 wt% at ambient pressure of carbon can be trapped in solid iron without forming the Fe_3C phase. Thus, the total carbon content has to be

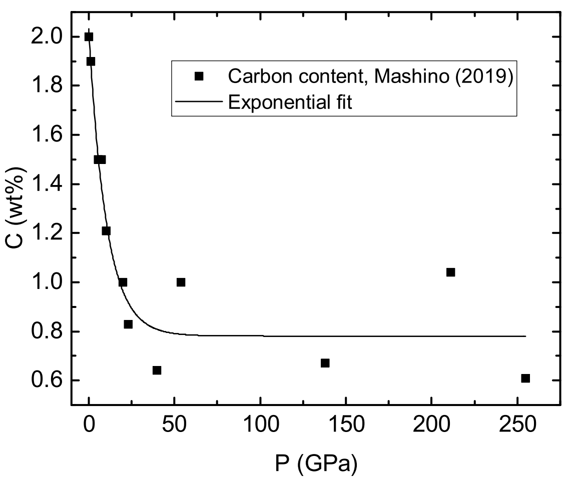

Figure 1: Carbon in solution in solid iron, as reported in Mashino et al.[1]. In first approximation, an exponential fit well describes the trend of the data with pressure.

calculated as the sum of the one contained in the Fe_3C phase and the one in solution in the iron phase.

The weight of carbon in solution in solid iron has been evaluated in several works at different pressures, as reported in Mashino et al. [1]. In first approximation, an exponential fit of the data well describes the trend of the carbon content as a function of pressure, as shown in Figure 1. The carbon weight found in Fe-C systems with LCA analysis, as reported in Figure 2, was thus added for the different pressures to the carbon weight resulting from the fit.

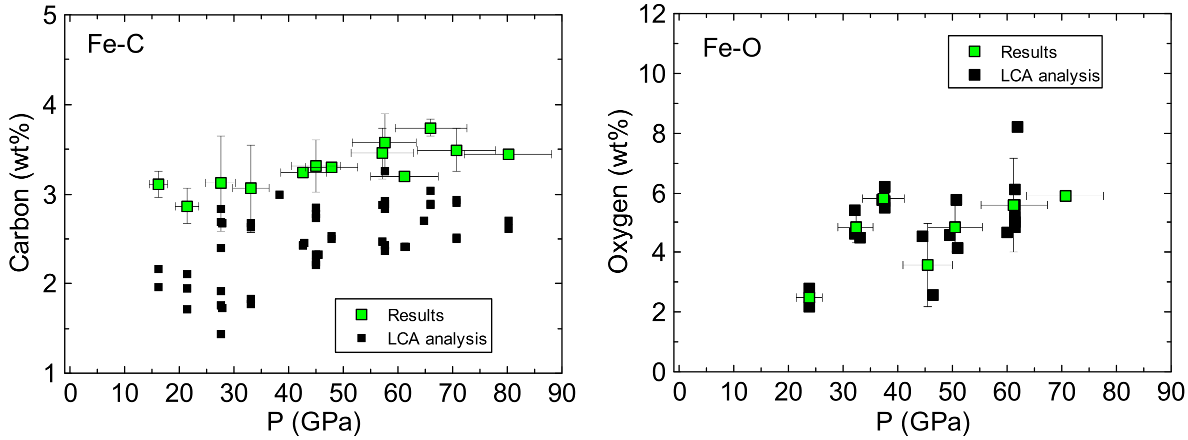

Figure 2: Raw results from LCA analysis are shown in black for Fe-C and Fe-O systems. Averaged total light element content is shown in green.

- Temperature effects

In some cases, after melting the light element content keeps slightly varying. Being unable to determine unquestionably which of those compositions is the eutectic, we averaged all of them defining as the error bar the variance of those points.

- Pressure effects

Both the quenched spectra and the references slightly vary with pressure. As the effect of pressure is to reduce the volume, XAS oscillations shift to the right. Thus it is important to use reference spectra whose pressure is as close as possible to the one of the spectrum under analysis. In case of doubt, reference spectra at the closest pressures were used and the results are averaged. Again the error bar is defined as the variance of the averaged points.

Results

In Figure 2 are represented in black the raw light element contents as obtained from the LCA analysis. In green are shown the final results, where the carbon in solution in solid iron is taken into account and the fit outputs at the same pressures are averaged. The error bar in the light element content is given by the standard deviation of the fit outputs at the same (or similar) pressure. The x-axis error bar is evaluated as 10% of the pressure value.

References

[1] Izumi Mashino, Francesca Miozzi, Kei Hirose, Guillaume Morard, and Ryosuke Sinmyo. Melting experiments on the Fe3C binary system up to 255 GPa: Constraints on the carbon content in the Earth's core. *Earth and Planetary Science Letters*, 515:135–144, 2019.
